# Network-level functional connectivity alterations in chemotherapy treated breast cancer patients: a longitudinal resting state functional MRI study

**DOI:** 10.1186/s40644-020-00355-6

**Published:** 2020-10-16

**Authors:** Yun Feng, Yun Fei Wang, Li Juan Zheng, Zhao Shi, Wei Huang, Long Jiang Zhang

**Affiliations:** 1 Department of Medical Imaging, Jinling Clinical Hospital, Nanjing Medical University, 305 Zhongshan East Road, Xuanwu District, Nanjing, 210002 Jiangsu China; 2 Department of Medical Imaging, Medical Imaging Center, The Affiliated Huai’an No.1 People’s Hospital of Nanjing Medical University, Huai’an, 223300 Jiangsu China; 3 Department of Medical Imaging, Jinling Hospital, Medical School of Nanjing University, 305 Zhongshan East Road, Xuanwu District, Nanjing, 210002 Jiangsu China; 4 Department of Medical Imaging, Jinling Clinical Hospital, Southern Medical University, 305 Zhongshan East Road, Xuanwu District, Nanjing, 210002 Jiangsu China

**Keywords:** Breast cancer, Chemotherapy, Cognitive impairment, Functional connectivity, Resting-state functional magnetic resonance imaging

## Abstract

**Background:**

Previous studies have found abnormal structural and functional brain alterations in breast cancer survivors undergoing chemotherapy. However, the network-level brain changes following chemotherapy remain unknown. The purpose of this study was to investigate the dynamic changes of large-scale within- and between-network functional connectivity in chemotherapy-treated breast cancer patients.

**Methods:**

Seventeen breast cancer patients were evaluated with resting state functional MRI (rs-fMRI), neuropsychological tests and blood examination before postoperative chemotherapy (t0), one week after completing chemotherapy (t1) and six months after completing chemotherapy (t2). Nineteen age- and education level-matched healthy controls (HC) were also recruited. Independent components analysis (ICA) was performed to assess network component using rs-fMRI data. The functional network changes were then correlated with cognitive assessment scores and blood biochemical indexes.

**Results:**

One-way repeated measures ANOVA revealed significantly changed within-network functional connectivity in the anterior and posterior default mode network (ADMN and PDMN), left and right frontoparietal network (LFPN and RFPN), visual network and self-referential network. Post-hoc test showed that decreased within-network functional connectivity in ADMN, PDMN, LFPN, RFPN, SRN and central network one week after chemotherapy and increased six months after chemotherapy (all *P* < 0.05). As for the between-network functional connectivity, the PDMN- sensorimotor network connectivity showed the same tendency. Most of these within- and between-network functional connectivity changes were negatively associated with blood biochemical indexes and cognitive assessment scores (all *P* < 0.05).

**Conclusions:**

These results indicated that chemotherapy may induce widespread abnormalities in resting state networks, which may serve as a potential biomarker of chemotherapy related cognitive impairment, providing insights for further functional recovery treatment.

## Background

Chemotherapy-related cognitive impairments (CRCI) are common in non-central nervous system cancers which show multiple-domain cognitive deficits such as attention, executive function, learning ability, memory and information processing speed during or after chemotherapy [1, 2]. Although accumulating research had focused on these cognitive problems, the neural mechanism remains largely unclear. Cross-sectional [3–5] and longitudinal neuroimaging studies [6–9] have shown structural and functional alterations during or after chemotherapy, thereby providing neural substrates for CRCI. Brain activation and connectivity studies also showed that breast cancer survivors had abnormal brain activity and decreased neural network transfer efficiency from large-scale perspectives [10–12]. These damaged brain structural and functional areas partially overlapped, mostly located in the frontal and temporal lobes. This indicates that chemotherapy induced common cognitive deficits may be related to the large-scale abnormal brain activity/connectivity.

Human cognitive activity is the result of the synergetic action of multiple functional neural networks in the brain, not only from the activities within the network, but also from the synergetic activities between the networks [13, 14]. Resting-state functional MRI (rs-fMRI) evaluates the resting state brain functional connectivity and makes it possible to describe the interaction of sub-networks, which are spatially distinct regions [15], and facilitates the understanding of the cooperative relationship of different brain regions at a large scale. Studies have found about 7 common resting state networks (RSNs) in healthy adults [16]. Alterations in RSNs have been observed in many neuropsychiatric diseases such as Parkinson’s disease, mild cognitive impairment and dementia [17–19]. Default mode network (DMN) is the most commonly investigated RSN in breast cancer survivors for its preferential vulnerability and sensitivity to tumor chemotherapeutics [20]. Decreased functional connectivity of DMN has been reported particularly in the medial prefrontal cortex, posterior cingulate cortex and the medial temporal cortex [15, 21]. Our previous study found abnormal hippocampal connectivity in superior/middle temporal gyrus, insula and frontal gyrus, most are parts of the RSNs [22]. These altered functional connectivities were involved in the regulation of executive, memory and emotion, supporting chemotherapy induced widespread cognitive networks disruption.

Most of the previous CRCI-related rs-fMRI researches focused on a specific RSN alteration in breast cancer patients [23, 24]. To the best of our knowledge, no studies have reported the dynamic changes of RSN functional connectivity in breast cancer survivors from a large-scale network perspective. Therefore, the purpose of this study was to investigate the dynamic changes of within- and between- network functional connectivity in chemotherapy-treated breast cancer survivors. We hypothesized that chemotherapeutic agents not only induced extensive RSNs abnormality, but also disrupted the connections between these RSNs. The correlations between changes in RSNs and potential risk factors such as anxiety, depression, hemoglobin and blood lipids, as well as cognitive assessment scores were also analyzed.

## Materials and methods

### Participants

**Fig. 1:**
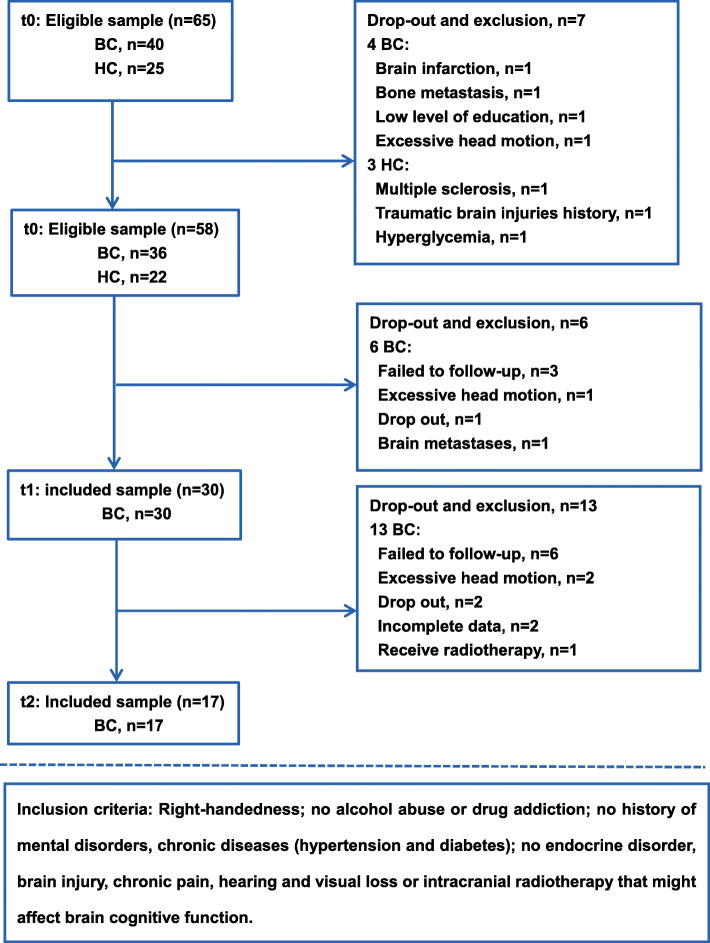
Flowchart of this study. BC, breast cancer; HC, healthy controls

The study was approved by the Medical Ethics Committee of Jinling Hospital (2016NZGKJ-069). All participants signed the written informed consent prior to the study. Women newly diagnosed with primary non-metastatic breast cancer (stages I-III) were recruited from March 2017 to December 2018. Brain MRI scans, cognitive assessment and blood biochemical examinations were performed at baseline (after surgery but before the start of chemotherapy or before neoadjuvant chemotherapy, t0), one week after completing chemotherapy (t1, mean 6 days), and six months after completing chemotherapy (t2, mean 171 days). Healthy controls (HC) recruited from the local community were evaluated at baseline. The inclusion criteria were: right handedness; with no history of psychiatric, neurological diseases and smoking; with 9 years or higher educational level; no chronic illness, no intracranial radiotherapy and other diseases that might affect brain function or cognitive assessment. Study flowchart with included and excluded patient numbers is shown in Fig. 1. Seventeen female breast cancer patients (mean age: 45.6 ± 10 years old) completing six to eight courses of chemotherapy and 19 age (mean age: 45.8 ± 9.4 years old) and education level matched HCs were finally included in this study (Fig. 1). There was no concurrent radiotherapy or endocrine therapy during chemotherapy for the 17 breast cancer patients, but 6/17 (35.3%) patients received endocrine therapy after the end of chemotherapy.

### Neuropsychological assessment

All participants completed the following neuropsychological assessments before each MR examination: 1) Mini-mental State Examination (MMSE) and Montreal Cognitive Assessment (MoCA), which were used to assess the participants’ common cognitive function; 2) Self-Rating Anxiety Scale (SAS) and Self-Rating Depression Scale (SDS), which were used to evaluate anxiety and depression state; 3) Number Connection Test type A (NCT-A) and Digital Symbol Test (DST) for the assessment of attention and executive ability; 4) Auditory verbal learning memory (WDT) for intelligence and memory assessment; 5) Line Tracing Test (LTT) for the visual ability; 6) Serial Dotting Test (SDT) for assessing the fine motor skills; 7) The Chinese version of the Stroop Color- Word Test (SCWT) for measuring reaction and conversion [21, 25]. All participants were scored according to the standard of each scale. The specific operation and instructions were completed by the same skilled researcher.

### Blood biochemical examinations

Fasting venous blood samples of all participants were obtained on the day of their MRI examinations to assess basic endocrine, immune and digestive function. Blood tests included Estradiol (E2), prolactin (PRL), follicle stimulating hormone (FSH), luteinizing hormone (LH), total cholesterol, triglyceride, high sensitivity C-reactive protein and hemoglobin (Hb) concentration. The fasting venous blood samples were obtained in the morning after sitting quietly for 15 min. For HCs with normal menstrual cycles, blood samples were collected 3–10 days after menstruation. All blood samples were collected, stored and tested in the same way.

### MRI data acquisition

Breast cancer patients underwent brain MRI scans at three time points on the same GE 3.0-T MRI scanner (Discovery MR 750, GE Healthcare, Milwaukee, USA) equipped with a standard 32-channel head coil. Our structural and functional MRI scan paradigms had been described in our previous study in detail [22]. The sagittal three-dimensional T1-weighted (3D-T1) images for anatomic reference were collected by using 3D inversion recovery prepared fast spoiled gradient recalled sequence. Repetition time (TR) = 8.14 ms, echo time (TE) = 3.17 ms, flip angle (FA) = 12°, matrix = 256 × 256, number of slices = 176 and slice thickness = 1.0 mm. Rs-fMRI data were acquired with a gradient echo EPI sequence during the motionless and unintentional thinking condition: total volume = 250, slices number = 10,750, TR = 2000 ms, TE = 30 ms, FA = 90°, matrix size = 64 × 64, fields of view (FOV) = 240 × 240 mm^2^. Routine T2- fluid attenuated inversion recovery (FLAIR) sequence was used to exclude primary brain lesion. The excluded primary brain lesions were brain infarction (*n* = 1) and multiple sclerosis (n = 1) at t0, brain metastases (n = 1) at t1 (Fig. 1). The HC group completed the MRI examinations at the baseline, follow-up examinations were not performed.

### Image preprocessing

The fMRI data analysis was done by using Data Processing Assistant for Resting-State fMRI (DPARSF) (http://rfmri.org/DPARSF). After removing the first 10 time points for the signal stabilization, a total of 240 time points were left for the following processing: slice timing correction was used to eliminate time errors between slices; realignment and head motion correction. Participants with head motion > 1.5 mm translation or rotation > 1.5° would be removed. Following realignment, there were 3 steps: 1) co-registration of functional images to the participants’ structural images; 2) T1 structural images were bias-corrected, segmented into grey matter, white matter and cerebrospinal fluid, and initially normalized to standard Montreal Neurological Institute (MNI) space; 3) the normalization parameters obtained were then applied to the functional images to normalize them into MNI space, and resampled with a voxel size of 3 × 3 × 3 mm^3^, the final images were smoothed using a 6 × 6 × 6 mm Gaussian kernel at full-width at half-maximum.

### Independent component analysis (ICA)

Analyses were conducted using ICA with GIFT software (http://icatb.sourceforge.net/). ICA is a data-driven, multivariate approach without priori assumptions which can separate unknown mixed fMRI signal sources into maximum spatial activation maps or independent temporal components. Independent components were estimated by GIFT software from pre-processed data of t0, t1 and t2 time points in the breast cancer group and HC group at baseline. Principal component analysis (PCA) was used to reduce the data dimension. Using the minimum description length criteria, 39 independent components were separated from four groups of smooth data dimensions [26]. We selected the ICASSO algorithm as stability analysis type by running ICA estimation 100 times. Subsequently, the Z value of each independent component was obtained by back-reconstruction using spatial-temporal regress. The z-value measured the correlation between the time series of each voxel and each independent component, which was used for further statistical analyses. Finally, the time series of 39 independent components reflecting the spontaneous brain activity and the spatial activation distribution map reflecting the intensity of brain activity were obtained. According to the maximum spatial correlation, the normalized average spatial activation map and the interested spatial template were calculated using automatic template matching process in the GIFT software. The component with the best “goodness of fit” of standard RSNs template was taken as the final resting state sub-network map [27]. In this study, 9 sub-network templates of RSN were used: default mode network (DMN), frontoparietal network (FPN), dorsal attention network, (DAN), sensorimotor network (SMN), central executive network (CEN), self-referential network (SRN), visual network (VN), auditory network (AN), and central network (CN).

### Statistical analyses

#### Demographic and clinical data

SPSS17.0 software (SPSS Inc. Chicago, IL) was used for the evaluation of the demographic and clinical data of all participants. Continuous variables were calculated the medians and the 25–75% interquartile ranges (IQRs). At baseline, the two-sample t-test was used to examine the differences between the breast cancer group and HC group. Non-normal distribution data were analyzed using two independent sample nonparametric tests (Mann-Whitney U method). One-way repeated measures ANOVA was used to test the within participants’ effects in breast cancer group, time as repeated effect, clinical data as the dependent variables. The *P* value was corrected by the Greenhouse-Gesisser correction method when it failed the spherical test. Bonferroni post hoc test was used to conduct pairwise comparisons between groups (t0, t1, t2).

#### Within–network functional connectivity

SPM8 software was used to obtain the group-level spatial distribution map of each RSN subnetwork by using the one sample t-test, family-wise error (FWE) corrected (*P* < 0.05). In order to restrict the within–network functional connectivity differences, a group mask was also generated. (1) Two-sample t-test was used to compare between the patients and controls at baseline, the Gaussian Random Field theory correction (GRF) (voxel *P* value < 0.001, cluster *P* value < 0.05) was used for multiple comparisons. Parameters such as SAS, SDS, age, education level and gray matter volume that may affect functional connectivity were used as covariates. (2) One-way repeated measures ANOVA was used to assess the dynamic changes of within-network functional connectivity in breast cancer patients at three time points (GRF correction, voxel P value < 0.001, cluster P value < 0.05). In the ANOVA model, we used REST software (http://www.restfmri.net/) to extract the average time series of significantly different brain regions within the subject, and evaluated the changes at t0, t1, and t2. Pairwise comparisons based on three time points were performed in the breast cancer group (post hoc test, *P* < 0.05).

#### Between-network functional connectivity

ICA time series was used to analyze between-network connectivity and the functional connectivity coefficients between the pairs of RSNs were obtained using the GIFT toolkit. The functional connectivity coefficients of all pairs of sub-networks were analyzed and the correlation coefficients results were converted to z values. In the breast cancer group, one-way repeated measures ANOVA was used to test the between-network functional connectivity coefficients differences at three time points (Bonferroni multiple comparison correction, *P* < 0.05). Post hoc test was used to conduct pairwise comparisons between groups (t0, t1, t2), and *P* < 0.05 was considered to be statistically significant.

### Correlation analysis

According to the formula (Δ value = t2-t1), the z value changes of significantly different within-network connectivity and between-network connectivity of each participant were calculated. Pearson correlation analysis was used to analyze the correlation between brain function connectivity parameter changes and clinical index changes. P < 0.05 was considered to be statistically significant.

## Results

### Demographic and clinical data

A total of 17 breast cancer patients receiving chemotherapy completed all tests at three time points (t0, t1 and t2). Nineteen HCs with matched age and education level only completed baseline assessments (t0). In breast cancer group, 11 women had normal menstrual status at baseline, 10 of the 11 premenopausal women had amenorrhea (90.9%, 10/11) at t1, and one had irregular menstrual cycles (9.1%, 1/11). In the breast cancer group, 6 patients received endocrine therapy after the end of chemotherapy. Six patients (35.3%, 6/17) recovered their menstruation at t2.

At baseline, there was no significant difference in menstrual status, E2, LH, and FSH levels between the breast cancer patients group and HC group (all *P* > 0.05). The baseline anxiety and depression scores in the breast cancer group were significantly higher than those in the HC group, however, these scores were in the normal range (*P* = 0.012, *P* = 0.002; respectively). The total cholesterol, triglyceride and fasting blood glucose in the breast cancer group were higher than those in the HC group (all *P* < 0.05), and the scores of SDT, WDT and Stroop test were higher than those of HC group (all P < 0.05). Demographic data and clinical data at baseline are shown in Table 1.

**Table 1:** Demographic, Clinical, and Neuropsychological Data at Baseline

Variables	BC group (*n* = 17)	HC group(*n* = 19)	*P* value
**Age (years)**			
median ± (IQRs)	46 (40–50)	47 (36–52)	0.505^c^
**Education levels (years)**			
median ± (IQRs)	12 (9–14)	15 (11–16)	0.065^c^
**Premenopause, n (%)**	11 (64.7)	11 (57.9)	0.896^b^
**Menopause, n (%)**	6 (35.3)	8 (42.1)	0.734^b^
**Breast cancer stage, n (%)**			
I	4 (23.5)	NA	NA
II	7 (41.2)	NA	NA
III	6 (35.3)	NA	NA
**Chemotherapy regimen stage (I-III)**		NA	NA
Postoperative chemotherapy, (I-III)			
AC-T (eight cycles), n (%)	11 (64.7)	NA	NA
Neoadjuvant chemotherapy, (I-III)			
TEC (six cycles), n (%)	5 (29.4)	NA	NA
AC-T (eight cycles), (IIB)	1 (5.9)		
**Clinical data, median ± (IQRs)**			
SAS (score)	29.0 (27.0–31.75)	25.0 (22.8–26.0)	0.012^c^
SDS (score)	27.5 (24.5–32.0)	23.0 (22.0–27.0)	0.002^c^
E2 (pmol/L)	265.0 (112.3–391.8)	83.0 (42.0–199.0)	0.220^c^
FSH (IU/L)	6.9 (3.9–40.1)	26.8 (8.1–80.3)	0.021^a^
LH (IU/L)	7.3 (2.2–25.4)	21.6 (8.3–40.6)	0.501^c^
Hemoglobin(g/L)	124.0 (110.0–134.0)	131.0 (125.0–136.0)	0.334^c^
Total cholesterol (mmol/L)	4.9 (4.3–5.7)	5.2 (4.5–5.7)	0.037^c^
Triglycerides (mmol/L)	1.5 (0.9–3.0)	0.8 (0.6–1.7)	0.030^c^
Fasting glucose (mmol/L)	5.2 (4.7–7.2)	4.9 (4.6–5.2)	0.005^a^
**Cognitive performance, median ± (IQRs)**			
MMSE (score)	28.0 (27.0–29.5)	29.0 (28.0–30.0)	0.061c
DST (score)	49.5 (34.0–61.8)	62.0 (45.0–69.0)	0.408^c^
LTT (sec)	59.8 (46.1–83.2)	32.3 (24.2–38.1)	0.429^c^
SDT (sec)	37.5 (33.3–42.3)	30.7 (27.1–34.5)	0.050^a^
WDT (score)	24.0 (22.0–27.0)	27.0 (24.5–29.3)	0.010^a^
Stroop-C (sec)	15.3 (13.5–18.4)	13.8 (9.9–15.8)	0.005^a^
Stroop-W (sec)	22.0 (18.5–26.1)	16.0 (13.5–18.5)	< 0.001^c^
Stroop-I (sec)	31.5 (24.9–33.0)	24.3 (19.2–28.4)	0.003^c^

a: k-independent samples nonparametric tests; b: chi square test; c: two-sample t test

*Abbreviations*: *A* Doxorubicin, *C* Cyclophosphamide, *T* Docetaxel, *E* Epirubicin, *SD* standard deviation, *E2* estradiol, *FSH* follicle stimulating hormone, *LH* luteinizing hormone, *DST* digit symbol test, *MMSE* Mini-mental state examination, *LTT* line tracing test, *NCT-A* number connection test A, *SDT* serial dotting test, *SDS* Self-Rating Depression Scale, *SAS* Self-Rating Anxiety Scale, *Stroop-C* Stroop colour test, *Stroop-D* Stroop word test, *Stroop-I* Stroop interference test, *WDT* Auditory verbal learning memory

### Demographic and clinical data changes

One-way repeated measures ANOVA showed that E2 values were significantly different among three time points (F = 5.089; *P* = 0.012). Post-hoc tests showed significant decrease from t0 to t1 (*P* = 0.041), and a rising trend 6 months after chemotherapy. Pairwise comparisons showed significantly increased triglycerides from t0 to t1 (P = 0.041) and decreased triglycerides levels at t2 without statistical significance. The Hb levels decreased slightly from t0 to t1, and significantly increased from t1 to t2 (*P* = 0.003) (Table 2).

**Table 2:** Blood Examination and Cognitive Assessment Results in Breast Cancer Group at Three Time Points

Median ± (IQRs)				*P* value			*F* value	*P* value
Characteristic	t0	t1	t2	t0 vs t1	t0 vs t2	t1 vs t2		
E2 (pmol/L)	265.0 (112.3–391.8)	81.0 (52.0–125.0)	138.0 (84.5–195.0)	0.041	0.188	0.506	5.089	0.012^c^
FSH (IU/L)	6.9 (3.9–40.1)	70.1 (10.3–89.9)	64.6 (19.9–112.2)	0.031	0.385	0.965	2.958	0.075^c^
LH (IU/L)	7.3 (2.2–25.4)	34.5 (5.8–51.5)	7.6 (3.4–29.0)	0.662	0.926	0.156	3.344	0.106^c^
Hemoglobin (g/L)	124.0 (110.0–134.0)	121.0 (113.3–126.0)	133.0 (123.5–142.0)	0.989	0.325	0.003	3.876	0.075^c^
Fasting glucose (mmol/L)	5.2 (4.7–7.2)	5.4 (4.8–5.7)	5.4 (5.2–5.7)	0.677	0.523	0.832	0.215	0.808^a^
Total cholesterol (mmol/L)	4.9 (4.3–5.7)	5.0 (4.5–5.4)	4.9 (4.7–5.2)	0.745	0.732	0.990	0.076	0.927^c^
Triglycerides (mmol/L)	1.49 (0.9–3.0)	2.0 (1.44–3.70)	2.1 (1.90–2.70)	0.041	1.000	0.194	3.281	0.052^a^
SAS (score)	29.0 (27.0–31.8)	32 (32.0–35.0)	27.0 (23.3–34.8)	0.186	0.659	0.098	1.548	0.228^a^
SDS (score)	27.5 (24.5–32.0)	34.0 (33.0–34.0)	29.0 (24.0–41.5)	0.133	0.221	0.677	1.418	0.257^c^
Stroop-C (sec)	15.3 (13.5–18.4)	14.4 (12.9–15.5)	14.4 (12.9–15.5)	0.480	0.493	0.985	0.340	0.714^c^
Stroop-W (sec)	22.0 (18.5–26.1)	18.9 (17.1–20.1)	19.0 (14.4–19.6)	0.235	0.179	0.876	1.156	0.327^c^
Stroop-I (sec)	31.5 (24.9–33.0)	29.0 (25.0–34.7)	24.0 (20.0–42.0)	0.999	0.992	0.991	0.001	0.999^c^
NST (sec)	47.2 (39.8–62.0)	41.0 (39.5–51.5)	53.0 (40.0–71.0)	0.973	0.187	0.224	1.101	0.345^c^
DST (score)	49.5 (34.0–61.8)	54.0 (45.0–64.0)	59.0 (43.0–60.0)	0.798	0.855	0.672	0.093	0.912^c^
LTT (sec)	59.8 (46.1–83.2)	40.0 (28.2–48.5)	39.0 (30.8–56.0)	0.010	0.012	0.978	5.013	0.013^c^
MMSE (score)	28.0 (27.0–29.5)	29.0 (28.0–29.5)	27.5 (26.3–29.8)	0.431	0.301	0.879	0.619	0.545^c^
SDT (sec)	37.4 (33.3–42.3)	33.5 (30.9–38.7)	32.0 (27.8–36.9)	0.451	0.247	0.659	0.728	0.491^c^
WDT (score)	24.0 (22.0–27.0)	21.0 (17.5–27.5)	18.0 (11.5–22.5)	0.278	0.002	0.082	5.595	0.008^c^

a: k-independent samples nonparametric tests; c: two-sample t test

*Abbreviations*: *t0* baseline assessment, *t1* one week after chemotherapy, *t2* six months after chemotherapy, *SD* standard deviation, *E2* estradiol, *FSH* follicle stimulating hormone, *LH* luteinizing hormone, *DST* digit symbol test, *MMSE* Mini-mental state examination, *LTT* line tracing test, *NCT* number connection test, *SDT* Serial dotting test, *SDS* Self-Rating Depression Scale, *SAS* Self-Rating Anxiety Scale, *Stroop-C* Stroop colour test, *Stroop-W* Stroop word test, *Stroop-I* Stroop interference test, *WDT* Auditory verbal learning memory

Repeated measures ANOVA showed significantly different WDT and LTT scores (*P* = 0.008, *P* = 0.013, respectively), the breast cancer patients showed slightly decreased WDT scores from t0 to t1, and significantly decreased scores from t0 to t2 (*P* = 0.002); LTT scores decreased significantly at t1 (*P* = 0.010) and slightly lower from t1 to t2, while the difference was statistically significant from t0 to t2 (*P* = 0.012) (Table 2). The NST, SDT and Stroop-test scores fluctuated after chemotherapy, but the differences were not statistically significant. Anxiety and depression scores were increased from t0 to t1 and reduced from t1 to t2, but the difference was not statistically significant (Table 2).

### Spatial distribution of RSNs

Thirty nine independent components were separated from the temporally concatenated 4D population data. After automatic template matching and visual discrimination confirmation, networks with the best matching were selected as the interested network. They were anterior and posterior DMN (ADMN, PDMN), DAN, left and right FPN (LFPN, RFPN), SMN, CEN, SRN, VN, AN and CN. One sample t-test showed that both breast cancer group and HC group demonstrated a typical spatial distribution pattern of RSNs.

### Within-network functional connectivity

Independent sample t-test showed no significant difference in within-network functional connectivity between the breast cancer group and HC group at baseline (GRF corrected).

**Fig. 2:**
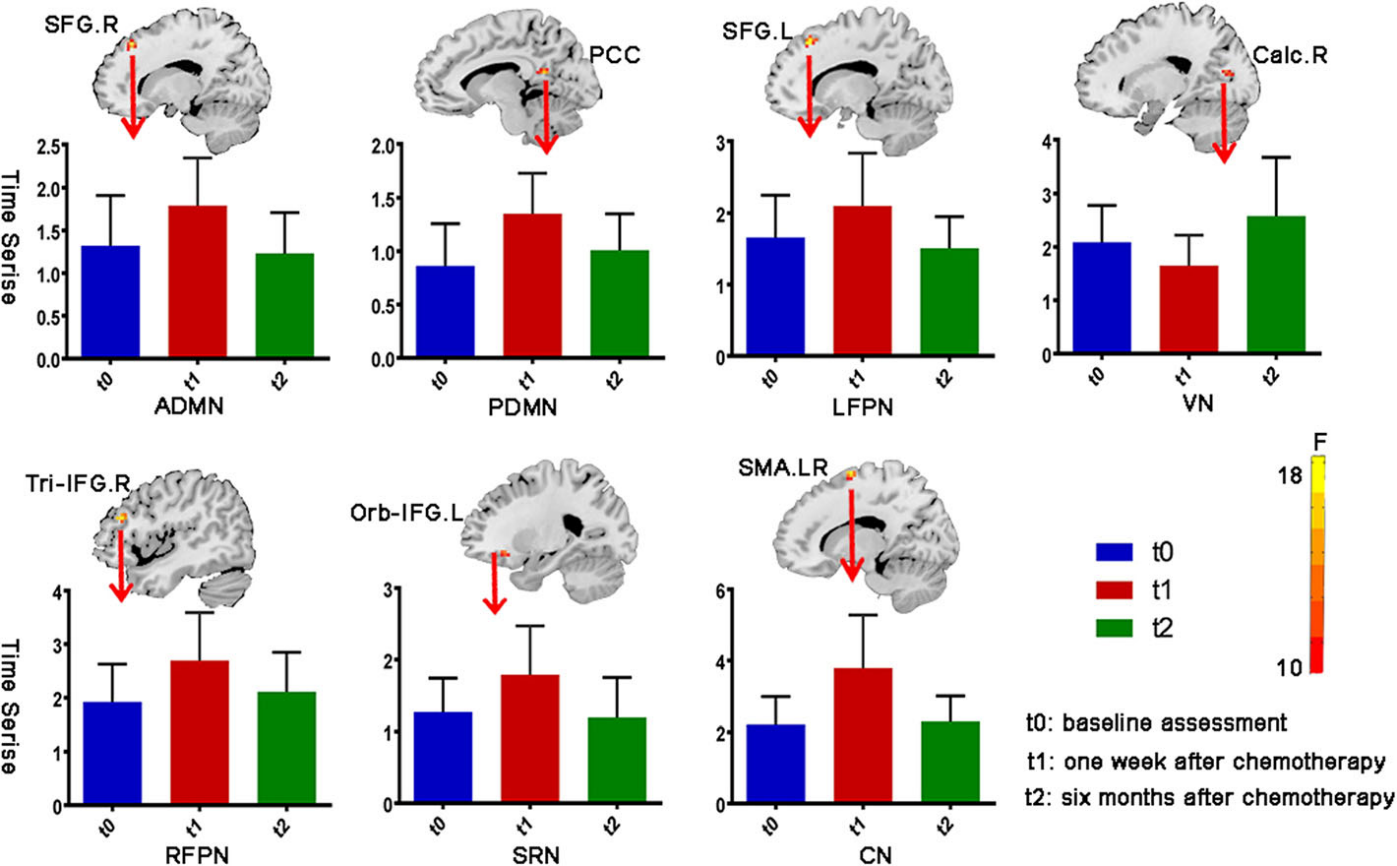
Seven RSNs show significant within-network connectivity changes in breast cancer patients among three time points. The histograms display the longitudinal evaluation of the within-network functional connectivity changes in the peak cluster regions for each RSN at three time points. t0, baseline assessment; t1, one week after chemotherapy; t2, six months after chemotherapy. SFG. R, right superior frontal gyrus, PCC, posterior cingulate gyrus, SFG. L, left superior frontal gyrus, Tri-IFG.R, Pars triangularis of right inferior frontal gyrus, SMA. R, right supplementary motor area, Orb-IFG.L, Left orbital inferior frontal gyrus, Calc. R, right calcarine sulcus cortex

The component of ADMN, PDMN, LFPN, RFPN, CN, SRN and VN revealed significantly different functional connectivity between time points in the breast cancer group (GRF corrected) (Tables 3, 4). The locations of the peak value cluster are shown in Table 3. Post-hoc tests showed significantly increased within-network functional connectivity from t0 to t1 in ADMN, PDMN, LFPN, RFPN, SRN and CN (*P* = 0.015, 0.001, 0.037, 0.006, 0.001 and 0.011, respectively) and significantly deceased connectivity in these above-mentioned networks from t1 to t2. Only the VN functional connectivity decreased slightly from t0 to t1 and significantly increased from t1 to t2 (*P* = 0.002) (Fig. 2).

**Table 3:** Statistically Significant Differences of Within-network Functional Connectivity for Breast Cancer Group

Anatomic region (RSN)	Cluster voxel	MNI Coordinates			F-score (peak value)
		x	y	z	
Right superior frontal gyrus (ADMN)	17	15	36	48	22.892
Posterior cingulate (PDMN)	13	−12	−51	18	16.763
Left supplementary motor area (CN)	50	16	1	70	16.752
Right supplementary motor area (CN)	30	−9	−3	69	16.047
Left superior frontal gyrus (LFPN)	27	−15	30	48	19.190
Pars triangularis of right inferior frontal gyrus (RFPN)	14	45	31	26	18.939
Left orbital inferior frontal gyrus (SRN)	8	−24	27	−9	15.689
Right calcarine sulcus cortex (VN)	14	18	−72	15	12.241

Gaussian Random Field theory correction, voxel *P* value < 0.001, cluster *P* value < 0.05

**Table 4:** Summary of Within-network Functional Connectivity for Breast Cancer Group at three time-points

	Median ± (IQRs)			*P* value			*F* value	*P* value
	t0	t1	t2	t0 vs t1	t0 vs t2	t1 vs t2		
ADMN	1.2 (0.7–1.8)	1.8 (1.4–1.8)	1.2 (0.8–1.7)	0.015	0.658	0.005	5.110	0.010
PDMN	0.8 (0.5–1.2)	1.2 (1.0–1.5)	1.0 (0.7–1.3)	0.001	0.270	0.012	7.321	0.002
LFPN	1.3 (1.2–2.0)	2.0 (1.6–2.3)	1.5 (1.1–1.9)	0.037	0.464	0.006	4.469	0.017
RFPN	2.0 (1.4–2.4)	2.5 (2.0–3.2)	2.0 (1.7–2.5)	0.006	0.489	0.034	4.518	0.016
CN	2.4 (1.4–2.9)	3.7 (2.5–4.9)	2.3 (1.7–2.9)	0.000	0.812	0.001	12.163	0.001
SRN	1.2 (0.9–1.7)	2.0 (1.2–2.2)	1.1 (0.7–1.7)	0.011	0.722	0.004	5.381	0.008
VN	2.1 (1.7–2.8)	1.5 (1.1–2.1)	2.2 (1.8–3.5)	0.123	0.087	0.002	5.503	0.007

t0, baseline assessment; t1, one week after chemotherapy; t2, six months after chemotherapy

### Between-network functional connectivity

**Fig. 3:**
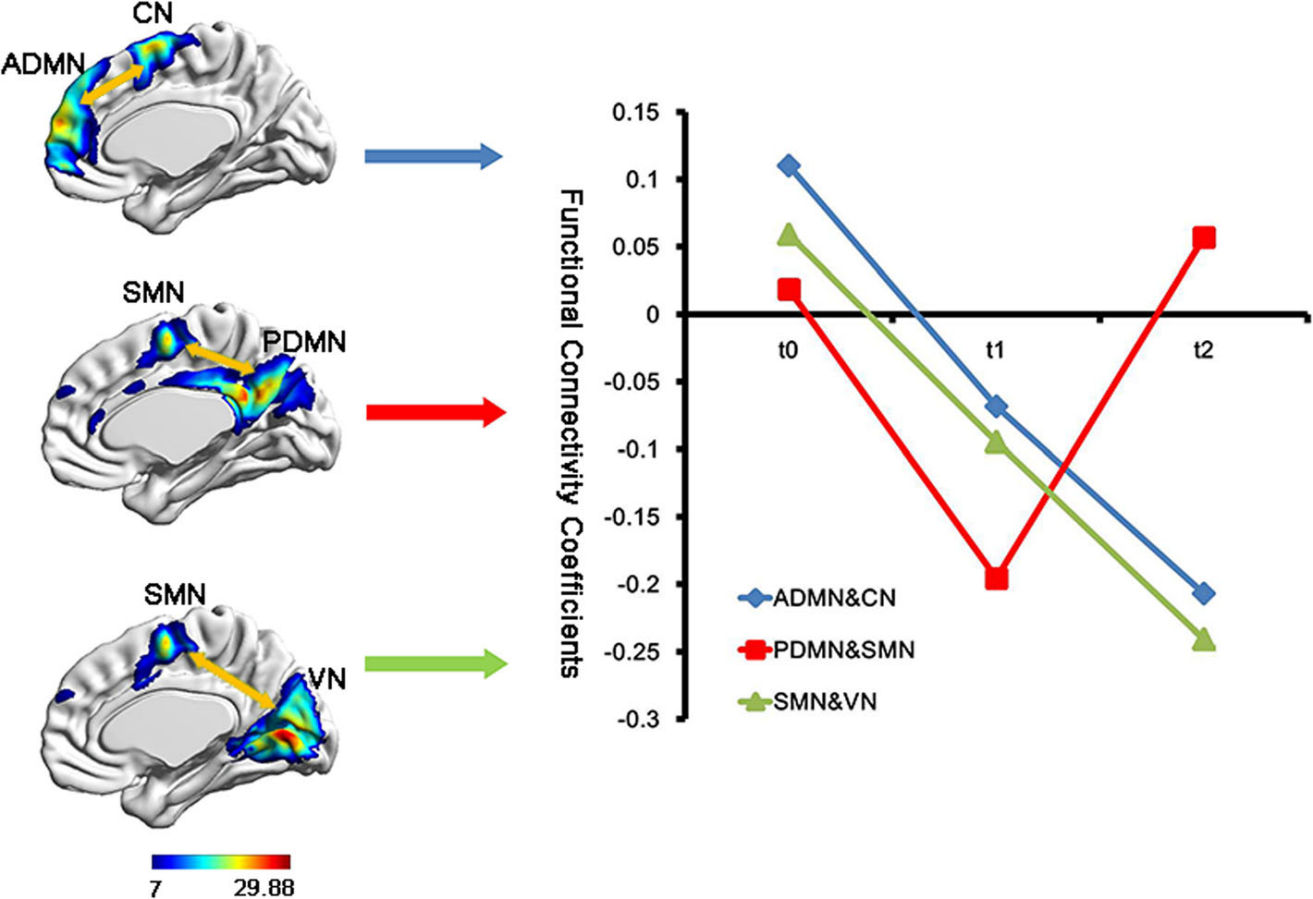
The between-network connectivity changes in breast cancer patients among three time points. t0, baseline assessment; t1, one week after chemotherapy; t2, six months after chemotherapy. ADMN, anterior default mode network; PDMN, posterior default mode network; SMN, sensorimotor network; VN, visual network; CN, central network

The between-network connectivity did not show significant difference between the patient and HC group at baseline. One-way repeated measure ANOVA analysis showed that the connectivity between ADMN and CN, PDMN and SMN, SMN and VN were different at three time points. Post-hoc test showed the functional connectivity between PDMN and SMN decreased from t0 to t1 (*P* = 0.051), while increased from t1 to t2 (*P* = 0.023). The functional connectivity between ADMN and CN, SMN and VN showed continuous decrease (all *P* < 0.05) (Fig. 3).

### Correlation analysis

**Fig. 4:**
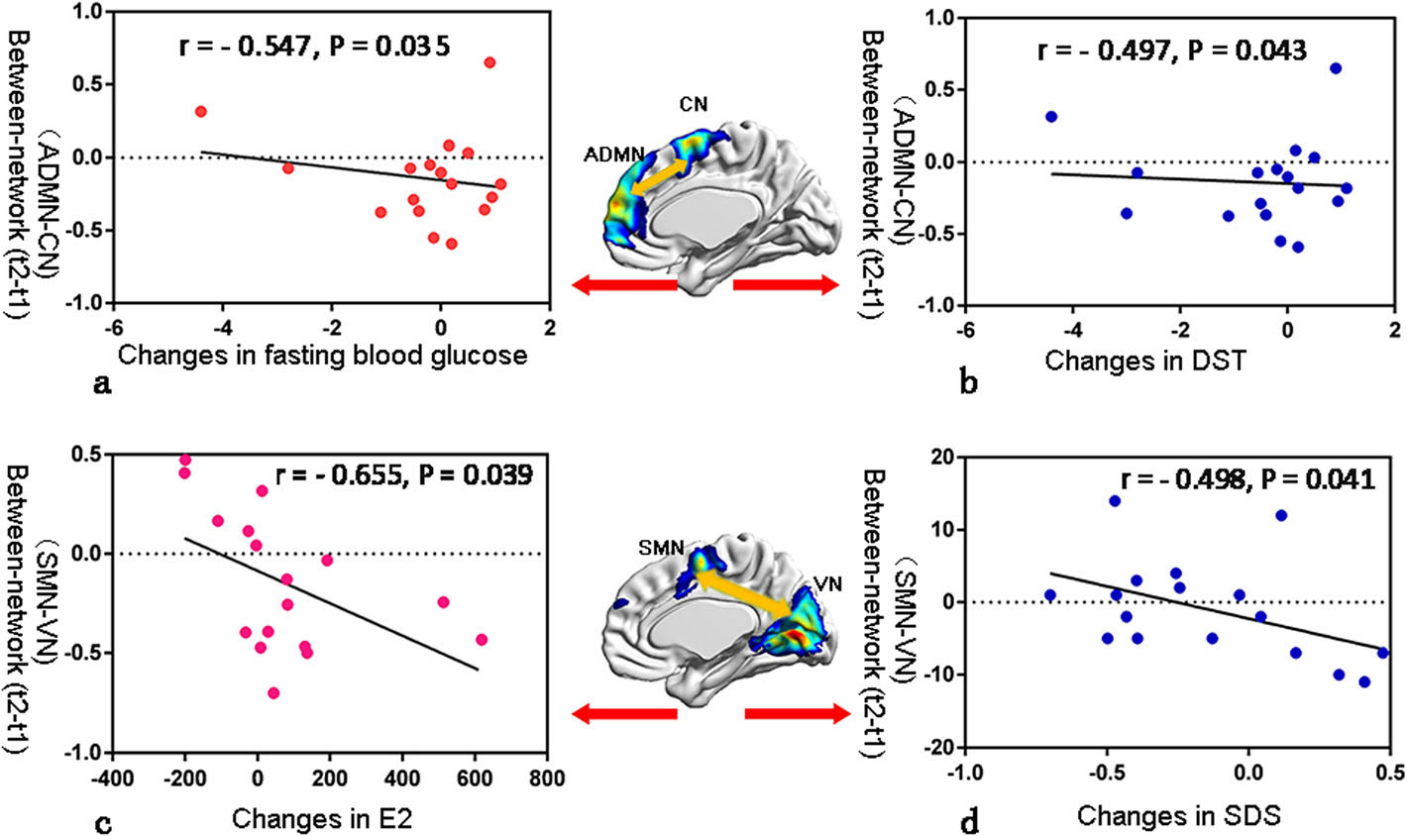
The correlation between functional connectivity changes and clinical variables changes in breast cancer survivors from t1 to t2. **a-b**: Correlation analysis of changes in the functional connectivity between ADMN and CN with changes in fasting glucose and DST scores. **c-d**: Correlation analysis of changes in the functional connectivity between SMN and VN with changes in blood estrogen levels and SDS scores. t1, one week after chemotherapy; t2, six months after chemotherapy. ADMN, anterior default mode network; SMN, sensorimotor network; VN, visual network; CN, central network; SDS, Self-Rating Depression Scale; DST, digit symbol test

The decreased between-network functional connectivity changes of ADMN and CN were negatively correlated with the decreased fasting glucose and increased DST score changes (r = − 0.497, *P* = 0.043; r = − 0.547, *P* = 0.035, respectively). The decreased between-network functional connectivity changes of SMN and VN were negatively correlated with increased changes in blood estrogen levels and decreased SDS scores (r = − 0.655, *P* = 0.039; r = − 0.498, *P* = 0.041, respectively) (Fig. 4). The decreased within-network connectivity changes in the supplementary motor area for CN were positively correlated with decreased fasting blood glucose changes (r = 0.561, *P* = 0.019) and negatively correlated with increased total cholesterol changes (r = − 0.484, *P* = 0.049). The increased within-network connectivity changes in the right calcarine sulcus cortex for VN were negatively correlated with the decreased changes of Stroop-W (r = − 0.563, P = 0.019).

## Discussion

In this study, we found increased within-network connectivity in most RSNs in breast cancer patients one week after chemotherapy, and all the within-network connectivity of RSNs tended to recover towards the baseline level six months after chemotherapy. Chemotherapeutics showed selective damage to the between-network connectivity. The connectivity between PDMN and SMN showed a recovery trend six months after chemotherapy, while the connectivity between ADMN and CN, SMN and VN continued to decline. Furthermore, both within- and between-network connectivity changes were significantly correlated with blood biomarkers and cognitive function alterations. This prospective longitudinal study provided the evidence that chemotherapy may induce widespread connectivity abnormalities in RSNs, which might serve as potential biomarkers of chemotherapy related cognitive deficits in breast cancer patients.

Our results suggested that chemotherapeutics can damage the large-scale resting state brain networks, including ADMN, PDMN, LFPN, RFPN, SRN, CN and VN. These findings provided the evidence that chemotherapy induced widespread cognitive deficits across various domains. DMN is one of the most common RSNs, which is considered to support processes such as active episodic memory and introspection, and would be deactivated during specific goal-directed tasks [28]. Previous studies have suggested that the ADMN is associated with emotional regulation, while the PDMN is involved in consciousness formation and memory [29, 30]. In the study, patients mainly exhibited memory impairment which may be associated with abnormal PDMN functional connectivity. CN is related to activity inhibition, playing an important role in adaptive cognitive control [16]. In this study, changes of ADMN-CN functional connectivity were negatively correlated with DST scores. This result indicated that dysregulated ADMN-CN may induce executive functioning and processing speed impairment. Interestingly, mind tracking and visual mobility showed improved in breast cancer survivors. We hypothesized that chemotherapy induced decreased VN and increased SMN functional connectivity may contribute to effectively coordinate this complex visual movement. However, we also found some slight but not significant cognitive impairment in attention, executive and reaction ability. The abnormal cognitive function may reflect brain mechanisms for attention and executive regulation involving DMN, CN and FPN [31].

In our study, chemotherapy induced abnormal RSNs functional connectivity was mainly located in the superior frontal gyrus, PCC, supplementary motor area, orbital inferior frontal gyrus and calcarine sulcus, which was consistent with the most previous studies [12, 20, 32]. We speculated that these regions may be more vulnerable to chemotherapeutic agent attack. PCC and superior frontal gyrus mainly located in the task-negative network and deactivated for self-referential processing tasks, while the supplementary motor area and orbital inferior frontal gyrus located primarily within or near the task-positive network and activated for demanding cognitive tasks. These different task-evoked neuronal responses were considered to support the process of target activity by regulating the allocation of neural resources [33]. We speculated that the increased functional connectivity may represent a compensation for chemotherapy induced cognitive dysfunction which required the recruitment of more neural regions and increased the strength of functional connectivity to maintain normal neural activity. Decreased functional connectivity in these regions may be attributed to the reallocation of neural resources in information processing [34, 35]. The functional connectivity strength of RSNs might predict chemotherapy induced higher or lower neural activity during task [30]. Previous multitask-based fMRI of CRCI showed increased [36–38] and decreased activity [39–41] located mainly in several frontal and parietal regions, anterior cingulate cortex and supplementary motor area. The functional connectivity regions of altered RSNs in our study were matched well with previous task-related studies.

Chemotherapeutics also showed selective damage to the between-network connectivity in this study. The DMN can integrate primary perception and advanced cognitive functions. It accepts more information from other RSNs than it outputs. Liao et al. showed that SRN, CEN, CN and AN had significant effect on connections to the DMN [42]. Sridharan et al. found that CN may regulate DMN, CEN and dorsal attention network [43]. The collaborative work among these RSNs completes the integration and transformation of information. In this study, the decreased functional connectivity between networks after chemotherapy suggested that chemotherapeutic drugs may alter function central brain regions through multiple inter-network connectives. The chemotherapy related cognitive impairments were the results of reduced coordination between multiple networks. The RSNs competed for more processing resources from the “central cognitive operator” to compensate for their impaired cognition [34]. This may explain the impairment of multiple cognitive domains after chemotherapy, but no significant difference was observed between baseline and post-chemotherapy.

Interestingly, most of the within-network connectives tended to recover to the baseline levels six months after chemotherapy, while the between-network connectives showed partial recovery. Recovery of some brain regions to baseline levels has also been reported in other CRCI-related functional and structural MRI studies [8, 44–46], and the acute damage mostly appeared 1 month after chemotherapy and (partially) recovered one year later. These studies suggested that chemotherapy-induced brain structural and functional damage may be temporary, the frontal regions (such as the frontal and temporal gyrus) may recover over time, and brain abnormalities in the posterior region may persist for a long time [47]. Brain function and structural recovery may be attributed to neuroplasticity mechanisms. In this study, most of the subjects were young women, cognitive challenges in daily work and social activities promoted early rehabilitation through neuroplasticity mechanisms. Additionally, the frontal regions began to show signs of recovery six months after chemotherapy. We hypothesized that the frontal lobe might be one of the first sensitive brain region to experience functional recovery. However, our finding was inconsistent with some previous studies as for recovery time, which may be related to the age, chemotherapy dose, chemotherapeutic drug, treatment stage, cognitive function type and the different control groups [1, 8, 48]. The recovery to the baseline level may reflect compensation ability improvements over time to some extent, it does not mean a return to normal level, and some brain dysfunction may persist for a long time. Sustained networks alterations may further affect patient’s cognitive function, such as sustained memory deterioration and executive ability impairment, which showed poor WDT and NST scores in this study. We are not sure whether the brain abnormality in the posterior brain region (such as visual function areas) alteration was a sustained change in acute effects after chemotherapy or a delayed brain injury occurring at a certain time after chemotherapy. Further long-term follow-up studies are thus needed.

Breast cancer patients also showed decreased visual ability and memory at baseline compared to HC group. This pre-treatment mild cognitive decline may be associated with tumor-related physical and psychological stress [41, 49]. Additionally, the improved test scores in LTT, SDT and Stroop test after chemotherapy may be related to the compensatory effect of the functional network. Furthermore, partial networks alterations were associated with estrogen, fasting blood glucose and blood lipids changes, though the improved blood indicator was still in the abnormal range compared to HC group. We speculated that the increased blood estrogen levels, decreased blood sugar and lipid levels six months later may play a positive role in the cognition improvement. Estrogen can alter the metabolic level of the frontotemporal lobe, affecting the structure and function of specific brain regions [50]. Animal experiments and clinical practice have shown that estrogen can be used to treat attention and memory loss. Abnormalities in blood lipids, fasting blood glucose, and hemoglobin have also been found in neuropsychiatric diseases such as Alzheimer’s disease and diabetic encephalopathy [51, 52]. The improved cholesterol metabolism would reduce the risk of vascular related cognitive impairment. However, the use of chemotherapeutic agents and endocrine therapy confused the assessment of underlying metabolic changes. Although there was no direct evidence that baseline metabolism changes were significantly associated with cognitive improvement six months after chemotherapy, we speculated that the improvement of baseline metabolism in patients can play an irreplaceable role in the recovery mechanism of CRCI.

Anxiety and depression scores were significantly higher in the breast cancer group than HC group at baseline, and continued to increase one week after chemotherapy, but decreased over time after the end of chemotherapy. The dynamic changes of depression and anxiety scores were similar as that of some cognitive functions. Studies had suggested that there were competitive interactions between emotion and cognition [7, 35], we speculated that the decreased negative emotion may benefit cognitive function recovery of patients in this study.

The study had some limitations. Although HC was included in this study, the control participants had no serial follow-up, making it impossible to evaluate the interaction between the groups and time. However, all breast cancer patients performed a series of neuropsychological tests, blood examination and MRI scans at three time points, which enabled the longitudinal analysis of RSNs in these patients. Secondly, the breast cancer patients were heterogeneous, some confounding factors such as different severities of the disease and different treatment strategies may induce some impacts on the results of our study. Thirdly, the basic metabolism and hormone level may fluctuate greatly in a relatively short time, and untangling these effects was difficult. Fourthly, the sample size is relatively small due to some patients refusing follow-up. Finally, memory effect can be present because of repeated neuropsychological testing at short time intervals, which may lead to no significant change and even improved in the cognitive test.

## Conclusion

This prospective longitudinal study found that the breast cancer survivors showed a large-scale functional connectivity damage after chemotherapy, and these impaired RSNs partly tended to return to the baseline level 6 months after chemotherapy. The altered functional connectivity was related to the patient’s cognitive function and hematology changes. Therefore, RSNs could be promising markers for evaluating potential chemotherapy-related brain damage, which provides an important basis for the observation of brain function impairment process and the subsequent cognitive rehabilitation interventions.

## Data Availability

The datasets used and/or analyzed during the current study are available from the corresponding author on reasonable request.

## Abbreviations

CRCI: Chemotherapy related cognitive impairment
fMRI: Functional magnetic resonance imaging
HC: Healthy controls
RSNs: Resting state networks
E2: Estradiol
DMN: Default mode network
FPN: Frontoparietal network
DAN: Dorsal attention network
SMN: Sensorimotor network
CEN: Central executive network
SRN: Self-referential network
VN: Visual network
AN: Auditory network
CN: Central network
PCC: Posterior cingulate cortex
